# Altered intestinal microflora and barrier injury in severe acute pancreatitis can be changed by zinc

**DOI:** 10.7150/ijms.45980

**Published:** 2021-06-16

**Authors:** Shi-yue Su, Qin-qing Tang

**Affiliations:** 1 Department of Obstetrics and Gynecology, the First Affiliated Hospital of Anhui Medical University, No.218 Jixi Road, Hefei 230022, Anhui, China.; 2 Department of General Surgery, the First Affiliated Hospital of Anhui Medical University, No.218 Jixi Road, Hefei 230022, Anhui, China.

**Keywords:** Endotoxin, IL-1β, intestinal microflora, pancreatitis, TNF-α, zinc

## Abstract

To investigate the effect of zinc (Zn) supplementation on intestinal microflora changes and bacterial translocation in rats with severe acute pancreatitis (SAP), the rats were divided into the sham surgery (SS), SAP, SS + Zn, and SAP + Zn groups. Saline (0.1 mL/100g) and 5% sodium taurocholate were injected into the pancreaticobiliary duct of the rats in the SS and SAP + Zn groups, respectively. Intraperitoneal injection of 5 mg/kg Zn was performed immediately after injecting saline or 5% sodium taurocholate into the rats in both groups. Serum amylase and Zn levels, plasma endogenous endotoxin, intestinal permeability, and the positive rate of intestinal bacterial translocation were detected, haematoxylin and eosin (H&E) staining was performed, and the pancreatic tissue scores were calculated for each group. In addition, immunohistochemical (IHC) staining was performed to evaluate the expression of IL-1β and TNF-α. Real-time fluorescence quantitative PCR was used to quantify the gene copy numbers of *Escherichia*, *Bifidobacterium,* and *Lactobacillus* in the cecum. The levels of amylase and plasma endotoxin in the SAP group were significantly higher than those in the SS and SS + Zn groups. Intestinal mucosal permeability and intestinal bacterial translocation in the liver, pancreas, and mesenteric lymph nodes were increased in the SAP group. However, the levels of amylase and plasma endotoxin were decreased as a result of zinc supplementation in the SAP group. The expression of IL-1β and TNF-α was also reduced to a greater degree in the SAP + Zn group than in the SAP group. Moreover, alleviated intestinal mucosal permeability and intestinal bacterial translocation in the liver, pancreas, and mesenteric lymph nodes were found in the SAP + Zn group. The results of real-time quantitative PCR showed that the gene copy number of *Escherichia* increased with time, and the gene copy numbers of *Lactobacillus* and *Bifidobacterium* decreased over time. Zn supplementation prevented the release of TNF-α and IL-1β, alleviated intestinal permeability and endotoxemia, reduced bacterial translocation, and inhibited changes in pathogenic intestinal flora in rats with SAP.

## Introduction

Approximately 80% of patients with acute pancreatitis (AP) have a good prognosis, with only minor symptoms. However, 20% of patients have a high mortality risk due to organ failure, with local and even systemic complications, which can lead to a high mortality rate of up to 50% 1, 2.

The intestinal mucosal barrier plays an important role in maintaining intestinal function and preventing bacterial and toxin transfer into the blood circulation in the enteric cavity. An intact gut mucosa serves as an effective barrier between the luminal bacterial microbiome, stool contents, and systemic circulation 3.

Intestinal bacteria are the main source of sepsis infections when the abdominal organs become inflamed. Bacteria may enter the body through the damaged mucosa and cause bacterial translocation 4. Severe acute pancreatitis (SAP) infection is considered to be the result of bacterial translocation (BT) from the gastrointestinal tract, and increased intestinal permeability has been implicated in this mechanism 5. BT is defined as endogenous bacteria (or its products) colonising the intestinal tract through the intestinal mucosal barrier and reaching the mesenteric lymph nodes and other distant sites 6.

Intestinal barrier function, which controls uptake across the mucosa and prevents damage by harmful substances from lumens, mainly consists of intestinal microflora, a mucus layer, the epithelium, intestinal circulation, mucosal immune system, and gut motility 7. A previous study demonstrated an increase in Gram-positive cocci, Gram-negative bacilli, and anaerobic microorganisms in the duodenum of animals with acute necrotising pancreatitis compared to the control group 8.

Zinc (Zn) is a trace element involved in signal transmission and immune response 9. Zn has antioxidant and anti-inflammatory properties, and maintains redox balance by regulating Zn-dependent enzymes, such as superoxide dismutase and matrix metalloproteinase 10, 11. Previous studies have shown that the levels of Zn in the SAP group were lower than those in the control group, and that Zn supplementation was beneficial to the balance of the free radical content, the antioxidant enzyme system of rats with SAP, and the liver and kidney 12, 13.

**Figure 1:**
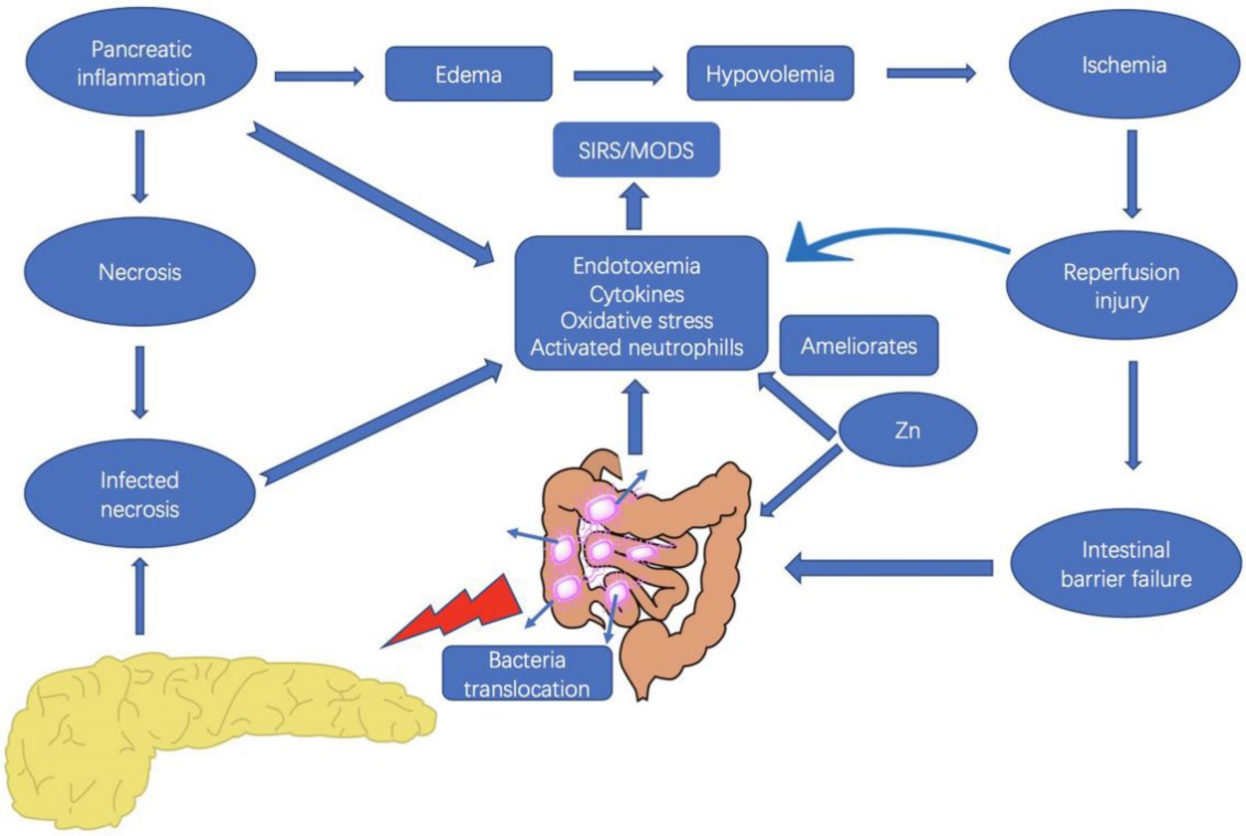
Interaction between the pancreas and the intestine, and the possible effects of zinc in severe acute pancreatitis. Zinc supplementation plays a role in the inhibition the release of inflammatory cytokines, as well as the reduction of bacterial translocation and endotoxemia.

However, the kinetics of functional impairment of the intestinal barrier in relation to pancreatic disease are unclear, and changes in the intestinal microflora have not been well elucidated. Therefore, we sought to investigate the SAP-associated bacterial translocation and intestinal flora changes, as well as IL-1β, TNF-α, and endotoxin levels in rats with SAP to determine the kinetics of intestinal barrier function impairment in Na-taurocholate-induced SAP models. Moreover, the possible role of Zn supplementation in modulation of IL-1β and TNF-α, bacterial translocation, and changes in pathogenic intestinal flora in the treatment of SAP were also explored (Fig. 1).

## Materials and Methods

### Materials

Sodium taurocholate was purchased from Sigma (USA). The Toxin Sensor^TM^ Endotoxin Kit was supplied by Kingsley Biotechnology Company (Nanjing, China). The Soil Genome DNA Rapid Extraction Kit and qPCR kit were obtained from Sangon Bio Company (Shanghai, China). Zn sulphate was obtained from the Tianjin Bodi Bio Company of China.

### Experimental animals

The experiments were conducted according to the guidelines of the committee of the Anhui Medical University of China. Seventy-two SD male rats weighing 200-300 g, provided by the Laboratory Animal Center of Anhui Medical University, were randomly divided into a sham surgery (SS) group (n = 18), SS + Zn group (n = 18), SAP group (n = 18), and SAP + Zn group (n = 18). Each group was further divided into 6 h (n = 6), 12 h (n = 6), and 24 h subgroups (n = 6). The rats were fasted overnight, except for free access to water before surgery.

All of the rats were anesthetized with 10% chloral hydrate (0.3 mL/100g) by intraperitoneal injection. A 1.5 cm incision was made in the mid-abdomen under aseptic conditions, and 5% sodium taurocholate reagent (0.1 mL/100g) was injected into the pancreaticobiliary duct. Saline (0.1 mL/100g) was injected into the pancreaticobiliary duct in the SS group. Both retrograde injections were administered into the pancreatic duct within 30 min using an automatic infusion pump. For the SAP + Zn group, 5 mg/kg Zn sulphate was administered by immediate intraperitoneal injection after a single injection of 5% sodium taurocholate. For the SS + Zn group, intraperitoneal injection of 5 mg/kg Zn sulphate was administered immediately after saline injection into the pancreaticobiliary duct.

Following closure of the abdomen, the rats were re-operated after 6, 12, and 24 h. Next, blood samples, mesenteric lymph nodes, liver, pancreas, ileum tissues, and cecum faeces were collected for further analysis.

### Determination of serum amylase, TNF-α, IL-1 β, endotoxin, and serum Zn

Serum amylase levels were analysed using an automatic HITACHI-7150 analyser (Japan). Briefly, 1 ml of inferior vena cava blood was placed into an anticoagulant tube and centrifuged for 15 min at 4000 rpm to separate the plasma. The endotoxin content in the plasma was determined using the endotoxin Limulus reagent from Nanjing Jiancheng BioCompany, China. The rats were treated in the same way in both the SAP + Zn and SAP groups after the administration of Zn sulphate 2 weeks later. One millilitre of inferior vena cava blood was collected with a syringe, and the serum was obtained by centrifugation (4°C, 15 min at 4000 rpm). Each blood sample was placed in an EDTA anticoagulation container to prevent clotting. The samples were then placed in a centrifuge to separate the serum at 3500 rpm for 5 min. The serum samples were stored at a temperature of -80°C until required for analysis. The serum Zn concentration was then determined using an atomic absorption spectrophotometer (Z-8100, Hitachi, Japan). The serum concentrations of TNF-α and IL-1 β were determined using ELISA (A&D Company of the United States). The experiments were performed in strict accordance with the manufacturer's instructions.

### Intestinal permeability assay

The small intestine (6 cm) was prepared and gently rinsed with saline. Subsequently, one end of the small intestine was closed, and the other end was ligated with a gavage needle. Then 0.2 ml Evans blue was injected with a 1 ml syringe into the intestinal tissue, and the processed intestinal tissue was placed in a 20-ml Krebs beaker in a 37°C water bath. The sac tissue was removed 30 min later, and the intestine was rinsed with saline until the rinse solution was clear. Next, the mixture was dried at 37°C for 24 h, 1 ml formamide solution was added, and the mixture was incubated at 50°C for 24 h. The solution was centrifuged, and the supernatant was analysed using an ultraviolet spectrophotometer at a wavelength of 620 nm. The Evans blue content (μg/g) was calculated according to the standard curve. The ultrastructure of the small intestinal mucosal epithelial cells was examined by transmission electron microscopy. Briefly, specimens of small intestinal mucosa were collected and placed on a precooled glass slide, and then 2.5% glutaraldehyde solution at 4°C was dripped rapidly onto the slide. The specimens were then cut into 0.5-1 mm² pieces and fixed in 2.5% glutaraldehyde solution at 4°C for 6 h. The ultrastructure of the small intestinal mucosa was then observed by transmission electron microscopy after double distilled water washing and natural drying.

### Histological evaluation and immunohistochemical studies

The sections of the pancreas and intestine were fixed with 4% paraformaldehyde and stained with haematoxylin and eosin (H&E). Based on the Spormann scoring system, pancreatitis can be classified into enema, haemorrhage, leukocyte infiltration, parenchymal necrosis, and adipose tissue necrosis according to the degree of severity 14.

Frozen and paraffin-embedded sections of the pancreas (5 μm) were subjected to standard avidin-biotin-peroxidase immunohistochemical methods. The sections were deparaffinized and rehydrated. The following antibodies were used: (1) rabbit anti-IL-1β polyclonal antibodies (1:50; Santa Cruz Biotechnology, Santa Cruz, CA, USA); and (2) mouse anti-TNF-α monoclonal antibody (1:100; Santa Cruz Biotechnology, Santa Cruz, CA, USA). The positive cells were determined by the presence of light brown granules in the cytoplasm. The distribution of positive cells was detected in non-overlapping fields in each section. The expression of IL-1β and TNF-α proteins was semi quantitatively analysed in 15 visual fields by calculating the average optical density (OD) of positive cells. Image-Pro Plus software (version 6.0) was used to perform semi-quantitative analysis on the expression of IL-1β and TNF-α, and to calculate the positive optical densities of IL-1β and TNF-α.

### Intestinal bacterial translocation assay

Under aseptic conditions, the lymph nodes, liver, and pancreatic tissues were ground sufficiently and placed in a 37°C incubator for nutrient requirement and anaerobic culture for 2 days before calculating the intestinal bacterial translocation rate.

### Extraction of DNA from cecum bacteria

Faecal samples (0.1 g) were mixed with 10 ml phosphate buffer for 5 min and then centrifuged for 10 min at 1000 rpm. Next, the bacterial genomic DNA was extracted from the supernatant and preserved at -20°C.

### Polymerase chain reaction (PCR)

Genomic DNA extracted from *Escherichia, Lactobacillus*, *Bifidobacterium*, and 16S rDNA was used for PCR amplification. The reaction system (Shanghai Sangon Biotech, Co., Ltd, China) consisted of 10 × PCR buffer 2.5 μl, 10 mmol/L dNTP mixture 0.5 μl, 10 μM upstream primers, 10 μM downstream primers, and 0.2 μl of Ex Taq DNA polymerase. Deionised water was added to the mixture to a final volume of 25 μl. The PCR amplification procedure was performed at 94°C for 5 min 30 s, annealing at 57°C for 30 s, and extension at 72°C for 8 min for a total of 35 cycles. Following amplification, 10 μl of the PCR product was taken, and 2% agarose gel electrophoresis was performed simultaneously with the DNA Marker.

### Construction of a standard plasmid

The amplified PCR products were purified and linked to the pMD18 simple-T vector. *Escherichia* Top10 was transformed, and four white clones were randomly selected. Next, the positive clones were screened according to blue-white selection and verified via PCR. A small amount of plasmid DNA was then prepared and identified by PCR and sent to Sangon BioCompany for sequencing. After accurate quantification using an ultraviolet spectrophotometer, the number of copies was calculated according to the following formula, diluted ten times, and then stored as a standard at -20 °C.

Copy number (copies/μl) = Concentration (ng/µl) × 6.02 × 10^14^/molecular weight

### Quantitative analysis of *Escherichia*, *Lactobacillus*, and *Bifidobacterium* by real-time quantitative PCR

The standard curve was established using real-time fluorescence quantitative PCR using a 10-fold gradient diluted plasmid standard as a template. The PCR reaction was performed in a 10 μl system, containing 5 μl Real Master Mix (SYBR Green), 1 μl template, 10 μM upstream primers, 10 μM downstream primers, and 3.6 μl deionised water. PCR amplification was performed at 95°C for 3 min, followed by 94°C for 15 s, annealing at 57°C for 20 s, and extension at 72°C for 30 s for 45 cycles. A standard curve was obtained. Genomic DNA samples of *Escherichia*, *Lactobacillus,* and *Bifidobacterium* were tested. The number of DNA copies in the samples was determined according to the measured Ct value and standard curve. The following primers were used: *Escherichia:* F: 5′-GTTAATACCTTTGCTCATTGA-3′, R: 5′-ACCAGGGTATCTTAATCCTGTT-3′; *Lactobacillus:* F: 5′-AGCAGTAGGGAATCTTCCA-3′ R: 5′-CACCGCTACACATGGAG-3′, *Bifidobacterium:* F: 5′-TCGCGTC(C/T)GGTGTGAAAG-3′ R: 5′-CCACATCCAGC(A/G)TCCAC-3′, 16S: F: 5′-ACGGGGGGCCTACGGGAGGCAGCAG-3′, R: 5′-ATTACCGCGGCTGCTGG-3'.

### Statistical analysis

Data were processed using SPSS software (version 17.0). The normality of the distribution was tested using the Kolmogorov-Smirnov test. Normally distributed data are expressed as the mean ± standard deviation (SD). Repetitive measure analysis of variance was used to analyse the influence of processing and time on each group, as well as the interaction between processing and time. The LSD-t method was used to compare the two treatments. P-values < 0.05 were considered statistically significant, and p-values < 0.01 were considered dramatic statistically significant.

## Results

### Lowering of the amylase levels caused by Zn supplementation in rats with SAP

**Figure 2:**
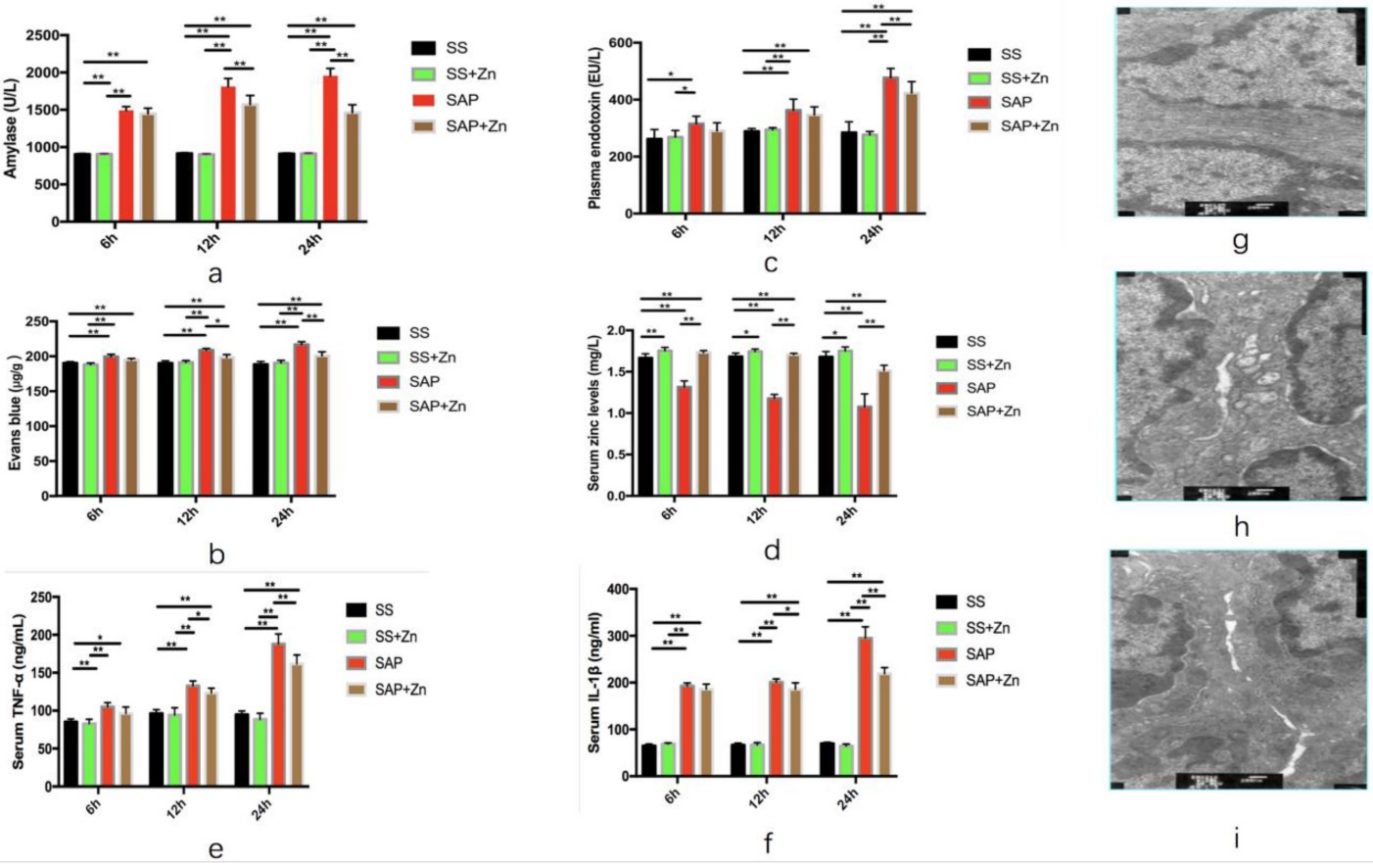
Levels of serum amylase, plasma endotoxin, and Evans blue were significantly increased, and those levels were markedly decreased in the SAP + Zn group compared to those in the SAP group. Levels of serum amylase in SAP + Zn group were significantly decreased compared to those in the 12 h and 24 h SAP group (**a**). Levels of plasma endotoxin in the SAP + Zn group were significantly decreased compared to those in the 24 h SAP group (**b**). Levels of Evans blue content in SAP + Zn group were significantly decreased compared to those in the 12 h and 24 h SAP groups (**c**). Levels of serum Zn levels in the SAP group were significantly decreased compared to those in the SS group and in the 6 h, 12 h, and 24 h SAP groups. However, the levels in the SAP + Zn group were increased compared to those in the SAP group (**d**). Levels of serum TNF-α and IL-1β in the SAP group were significantly increased compared to those in the SS group and SS + Zn group, and in the 6 h, 12 h, and 24 h SAP groups. However, the levels in the SAP + Zn group decreased significantly compared to those in the SAP group (**e, f**). The intercellular space was found to be widened in the SAP group (**h**) compared to the SS group (**g**), as observed by transmission electron microscopy. However, the injury was less severe in the SAP + Zn group (**i**) than in the SAP group. The data are expressed as means ± SD, *p < 0.05 and **p < 0.01.

Acute pancreatitis was diagnosed by elevation of the serum alpha-amylase level. The levels of amylase in the SAP group were higher than those in the SS and SS + Zn groups. Statistical analysis showed that there was a significant difference between the SAP and SS groups. However, Zn supplementation decreased amylase levels. In the subgroup analysis, the difference between the SAP and SAP + Zn groups was significant after 12 h and 24 h (Fig. 2a).

### Reduction in plasma endotoxin caused by Zn supplementation in rats with SAP

Plasma endotoxin levels in the SAP group were higher than those in the SS and SS + Zn groups. Statistical analysis showed that there was a significant difference between the SAP, SS, and SS + Zn groups. However, the difference between the SAP and SAP + Zn groups was only significant in the 24 h subgroup (Fig. 2b).

### Reduction of intestinal permeability caused by Zn supplementation in rats with SAP

The SAP group showed higher dye extravasation than the SS and SS + Zn groups on the basis of Evans blue exudation, showing that intestinal mucosal permeability was increased in the SAP group. The Evans blue content was significantly decreased in the SAP + Zn group 12 h and 24 h later than that in the SAP and SS + Zn groups (Fig. 2c).

### Increase in serum Zn levels in the SAP + Zn group caused by Zn supplementation when compared to rats with SAP

The serum Zn levels in the SAP group decreased significantly compared to the SS and SS + Zn groups, but increased in the SAP + Zn group compared to the SAP group (Fig. 2d).

### Reduction of serum TNF-α and IL-1β levels caused by Zn supplementation in rats with SAP

The TNF-α and IL-1β serum levels in the SAP group were higher than those in the SS and SS + Zn groups. However, the levels of TNF-α and IL-1β were significantly decreased in the SAP + Zn group 12 h and 24 h later than those in the SAP group (Fig. 2e and Fig. 2f).

### Inhibition of IL-1β and TNF-α release and alleviation of SAP severity by Zn supplementation according to histopathological examination and immunohistochemical staining

**Figure 3:**
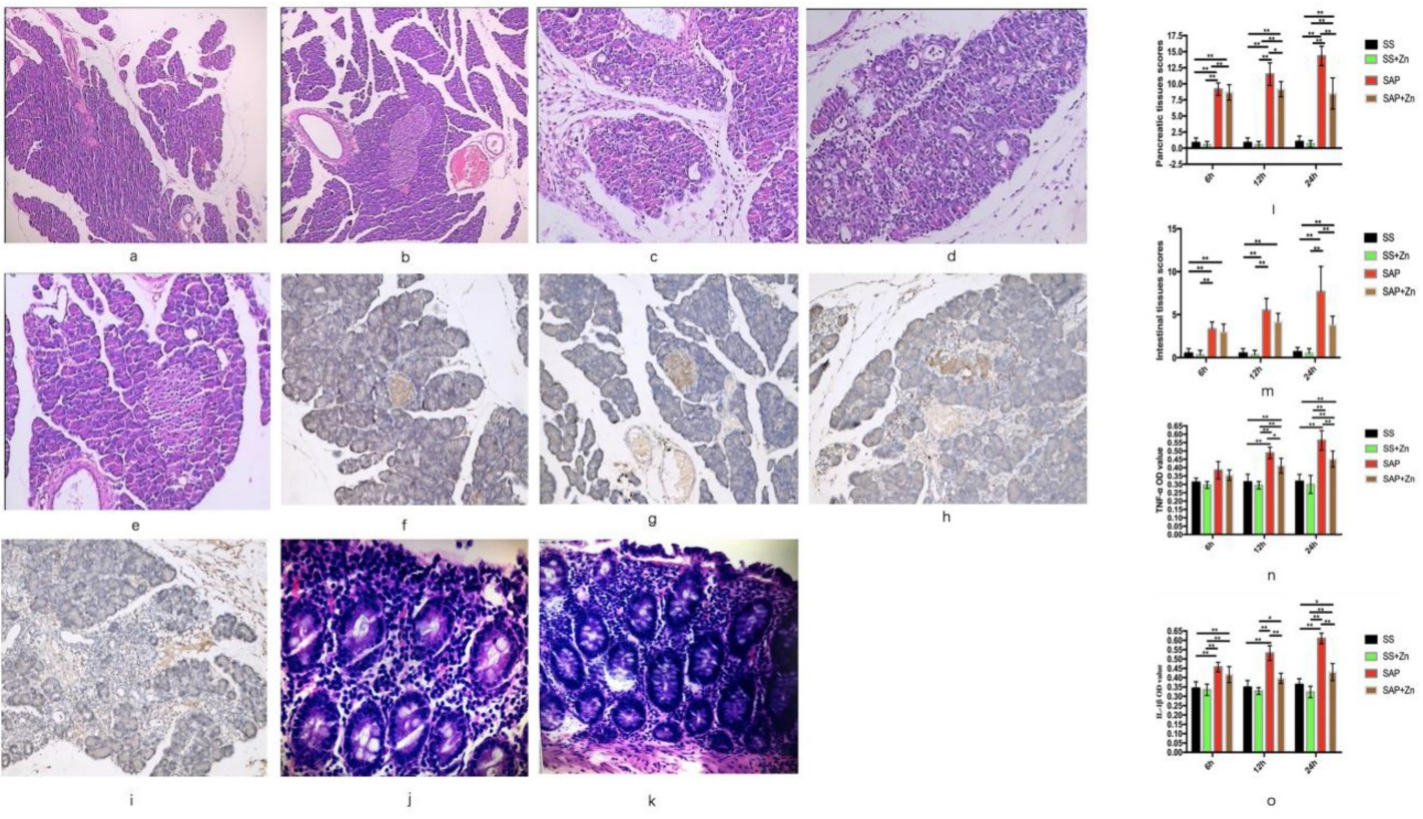
HE staining of the pancreatic tissue of the SS group (HE × 100), showing normal pancreatic tissue (**a, b**). HE staining of the SAP 24 h group, showing interstitial oedema and neutrophil infiltration into the pancreatic tissue (HE × 400) (**c, d**); and showing alleviated oedema and neutrophils infiltration in the Zn-treated SAP group (24 h) (HE × 400) (**e**). Immunohistochemical analysis of IL-1β expression in the SS group (× 400) (**f**); immunohistochemical analysis of TNF-α expression in the SS group (× 100) (**g**); immunohistochemical analysis of IL-1β expression in the SAP 24 h group (× 400) (**h**); immunohistochemical analysis of TNF-α expression in the SAP 24 h group (× 400) (**i**). HE staining of the intestinal tissue showed neutrophil infiltration into the intestinal tissue (HE × 400) (**j, k**). Pancreatic tissues scores of the SAP 6 h, 12 h, and 24 h groups; the scores increased with time. However, the pancreatic tissue scores decreased in the Zn-treated SAP group (**l**). Intestinal tissues scores of the SAP 6 h, 12 h, and 24 h groups; the scores increased with time. However, the intestinal tissue scores decreased in the Zn-treated SAP group (**m**). TNF-α OD value of the SAP 6 h, 12 h, and 24 h group; the OD values increased with time. The OD value was significantly decreased in the 12 h and 24 h Zn-treated SAP groups (**n**). IL-1β OD value of the SAP 6 h, 12 h, and 24 h groups; the OD values increased with time. However, the OD value significantly decreased in the 12 h and 24 h Zn-treated SAP groups (**o**). The data are expressed as means ± SD, *p < 0.05 and **p < 0.01.

Oedema, haemorrhage, and leukocyte infiltration were observed in the SAP group. The pancreatic tissue scores, as well as the positive optical densities (ODs) of IL-1β and TNF-α increased significantly over time. However, these scores and OD values were significantly decreased in the SAP + Zn group 12 h and 24 h later than those in the SAP group. HE staining of the intestinal tissue showed neutrophil infiltration into the intestinal tissue in the SAP group (Fig. 3).

### Zn leads to a significant reduction in translocated bacteria in the liver, pancreas, and mesenteric lymph nodes in comparison to the SAP group

**Figure 4:**
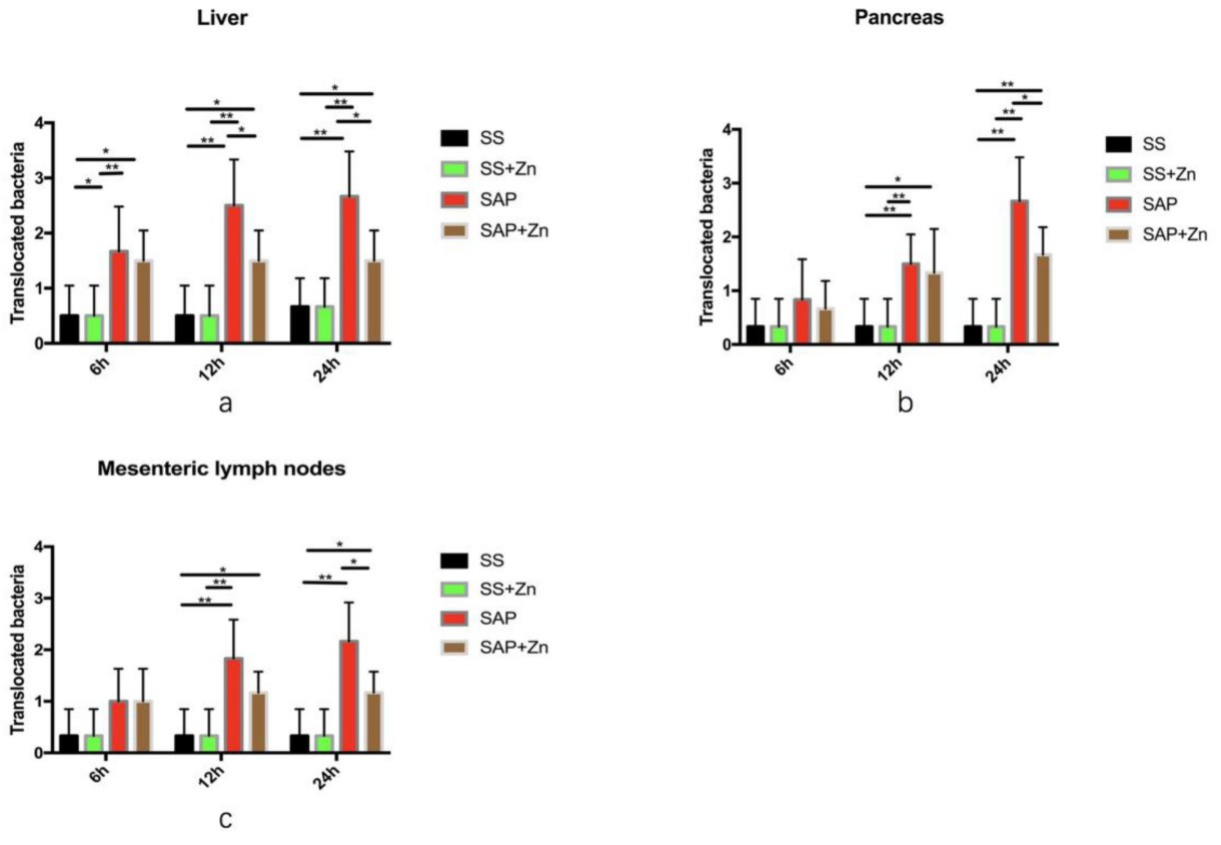
Translocated bacteria in the liver, pancreatic tissues, and mesenteric lymph nodes. Liver (**a**); pancreas (**b**); mesenteric lymph nodes (**c**). The data are expressed as means ± SD, *p < 0.05 and **p < 0.01.

The amount of translocated bacteria in the liver was much larger in the SAP group than in the SS and SS + Zn groups. However, the amount of translocated bacteria was significantly decreased in the SAP + Zn group after 12 h and 24 h compared to the SAP group (Fig. 4a). The amount of translocated bacteria in the pancreas and mesenteric lymph nodes was significantly higher in the SAP group 12 h and 24 h later than that in the SS group. Zn significantly decreased the amount of translocated bacteria compared to the SAP group (Figs. 4b-c).

### Reduction in gene copy number of *Escherichia,* and increase in *Bifidobacterium* and *Lactobacillus* in the SAP + Zn group compared to the SAP group

**Figure 5:**
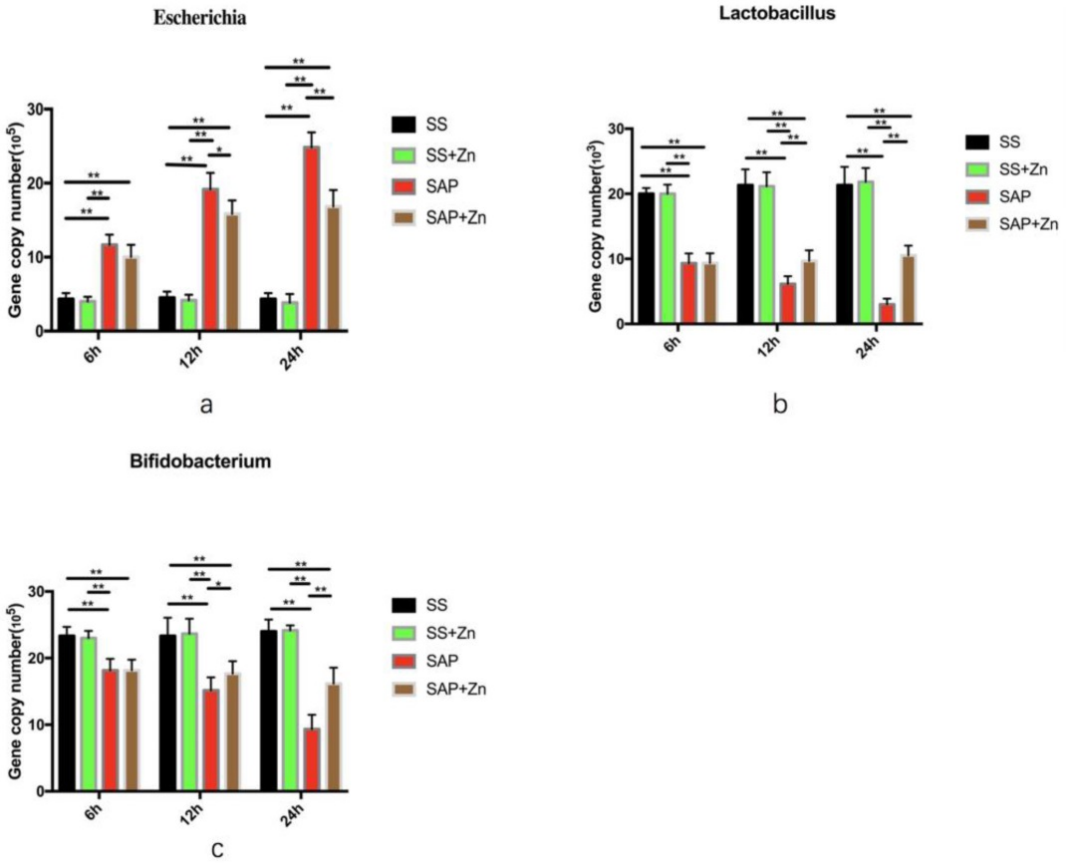
The gene copy number of *Escherichia* in the SS, SAP, and SAP + Zn groups (**a**). The gene copy number of *Lactobacillus* in the SS, SAP, and SAP + Zn groups (**b**). The gene copy number of *Bifidobacterium* in the SS, SAP and SAP + Zn groups (**c**).

Samples isolated from the cecum were analysed. The dissolution curves of the three species of bacteria and internal reference were generated, with temperature as the abscissa. The dissolution curves showed a single peak, which demonstrated the specificity of the primers. The absolute quantification of the three species of bacteria showed that the gene copy number of *E. coli* in the SAP group was significantly higher than that in the SS and SS + Zn groups, while the gene copy numbers of *Bifidobacterium* and *Lactobacillus* were significantly lower than those in the SS and SS + Zn groups. Additionally, the gene copy number of *Escherichia* increased with time, while those of *Lactobacillus* and *Bifidobacterium* decreased. However, the gene copy number of *Escherichia* decreased, and *Bifidobacterium* and *Lactobacillus* increased in the SAP + Zn group compared to those in the SAP group (Fig. 5 and Fig. S1). The data are expressed as the mean ± SD, *p < 0.05 and **p < 0.01.

## Discussion

Bacterial translocation is defined as the translocation of intestinal bacteria from the intestinal cavity to the mesentery or other extraintestinal organs, including mesenteric lymph nodes (MLNs) and distant organs 15. Many studies have confirmed the presence of bacterial translocation in critically ill patients and their relationship with the development of sepsis 16-19. According to previous studies, bacterial translocation is widely accepted as the main source of systemic infection and may play an important role in the pathogenesis of sepsis in critically ill patients 20, 21. Multiple organisms are commonly observed in the blood specimens of patients with SAP using denaturing gradient gel electrophoresis 22. However, the changes in the intestinal microflora in SAP remain unclear.

Changes in the intestinal flora are related to the pathogenesis of several inflammatory disorders 23. However, evidence for the role of intestinal flora changes in SAP remains insufficient due to the limited number of studies discussing changes in intestinal microbiota in patients with SAP. *E. coli* is the most common bacteria associated with AP infections 24. Wu et al. showed that the amount of *Bifidobacteria* and* Lactobacillus* decreased, while the amount of *E. coli* increased in patients with acute pancreatitis accompanied by concurrent infection. *Bifidobacteria* play a role in the prevention of pancreatic infections 25. Tan et al. found that the amount of *Bifidobacterium* and *Lactobacillus* decreased in patients with SAP compared to that observed in patients with mild acute pancreatitis 26.

In this study, significant changes in microbial composition in the cecum were observed between the SAP group and the SS and SS + Zn groups. In the SAP group, Escherichia increased significantly, whereas *Bifidobacterium* and *Lactobacillus* decreased significantly. Thus, our research is in accordance with previous studies. *Bifidobacterium* and *Lactobacillus* have many beneficial effects on the stabilisation of intestinal barriers. The reduction of these beneficial bacteria and the increase of potentially pathogenic bacteria, such as Escherichia, correlates with intestinal flora disorder and SAP progression.

The main function of Zn is to act as a cofactor for the enzyme or structural element of the protein. Zn deficiency can result in ineffective control of pathogens and intestinal barrier dysfunction 27, 28, 29. Some animal models have shown that Zn supplementation can reduce the severity of sepsis 30, 31. Reed et al. demonstrated that microbes in the gut affect Zn levels 32. The availability of Zn affects intestinal flora 33, and Zn deficiency is common in patients with CP 34. However, studies concerning the Zn status in patients with acute pancreatitis are limited.

Through our research, Zn was found to alleviate intestinal permeability, bacterial translocation, endotoxemia, and the severity of SAP. The composition of the intestinal microbiota can also be altered by Zn in the progression of SAP. The expression of IL-1β and TNF-α was significantly decreased in the SAP + Zn group. The above discussion indicates that Zn affects the composition of intestinal microbiota and inhibits the progression of SAP.

Zn homeostasis disorders in patients with pancreatitis are related to the progression of the inflammatory process 35. Osman et al. demonstrated that Zn is useful in AP because it eliminates harmful enzymes from circulation by increasing the stimulation of 2-macroglobulin synthase and RES 36. Low intracellular Zn levels have also been found to be associated with DNA damage, oxidative stress, antioxidant defence, and DNA repair, and reduced Zn is a potential marker for the treatment of resistant depression and the immune/inflammatory response in depression 37. There is also evidence that Zn is an important antioxidant 38. Oxidative stress is characterised by an imbalance between free radical exposure and antioxidant defence, which includes small molecular weight antioxidants, such as metallothionin and antioxidant enzymes. Zn supplementation alleviates oxidative stress, which may be a possible mechanism for maintaining gut barriers and reducing the release of cytokines and bacteria into the bloodstream in rats with SAP.

Zn plays an important role in intensifying the immunological response and has a protective effect on BT. Moreover, Zn deficiency is associated with increased systemic inflammation and increased nuclear factor-kB (NF-kB) activity 39, 40. Zn supplementation has anti-inflammatory effects, reduces intestinal permeability, and increases villus height, Villin expression, and the amount of intestinal brush border enzyme 41, 42. Hence, Zn supplementation alleviates inflammation, which may be another possible mechanism for maintaining gut barriers and reducing the severity of SAP.

## Conclusions

The results of this study showed that intestinal bacteria translocated, and the composition of intestinal bacteria changed in the SAP group. Zn prevented the release of TNF-α, IL-1β, and bacteria into the bloodstream, reducing bacterial translocation, and inhibiting changes in the pathogenic intestinal flora, which may be important in the treatment of SAP. Maintaining the gastrointestinal microecological balance may be an effective way to regulate SAP progression, and supplementation with Zn is one possible mechanism.

## Supplementary Material

Supplementary figure.
