# Supplementary figures and images for: Altered intestinal microflora and barrier injury in severe acute pancreatitis can be changed by zinc
Source: Int J Med Sci. 2021 Jun 16;18(14):3050–8. doi: 10.7150/ijms.45980 (PMC8364456; doi:10.7150/ijms.45980)

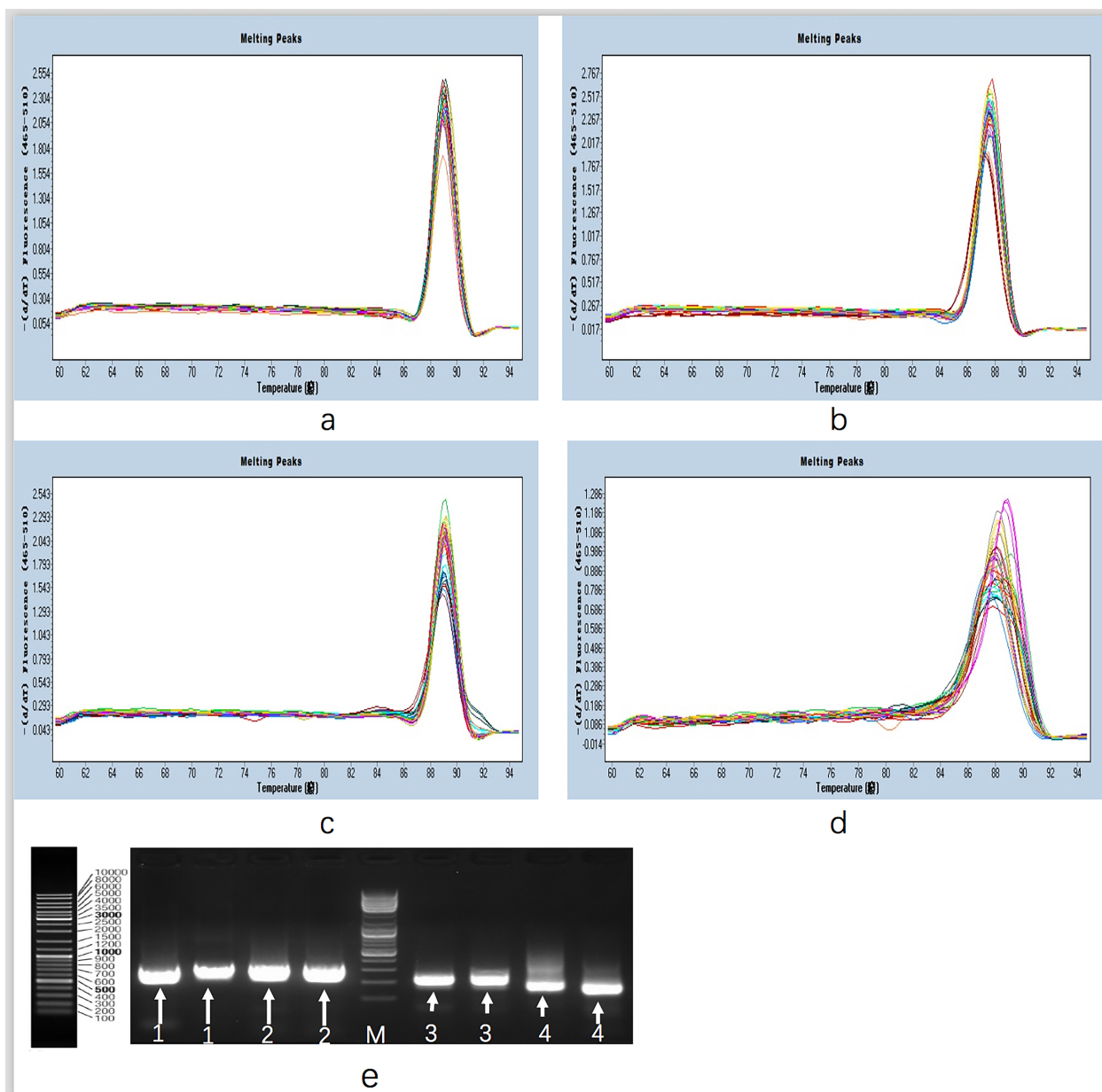

Figure S1

Supplement: Supplementary file 1 — Supplementary figure. [file ijmsv18p3050s1.pdf]
